# An international survey on AI in radiology in 1,041 radiologists and radiology residents part 1: fear of replacement, knowledge, and attitude

**DOI:** 10.1007/s00330-021-07781-5

**Published:** 2021-03-20

**Authors:** Merel Huisman, Erik Ranschaert, William Parker, Domenico Mastrodicasa, Martin Koci, Daniel Pinto de Santos, Francesca Coppola, Sergey Morozov, Marc Zins, Cedric Bohyn, Ural Koç, Jie Wu, Satyam Veean, Dominik Fleischmann, Tim Leiner, Martin J Willemink

**Affiliations:** 1 Department of Radiology, University Medical Center Utrecht, Utrecht, The Netherlands; 2 Department of Radiology, Elisabeth-TweeSteden Ziekenhuis, Tilburg, The Netherlands; 3 Department of Radiology, University of British Columbia, Vancouver, Canada; 4 Department of Radiology, Stanford University School of Medicine, Stanford, CA USA; 5 Department of Radiology, Motol University Hospital, Prague, Czech Republic; 6 Department of Radiology, University Hospital of Cologne, Cologne, Germany; 7 Department of Radiology, IRCCS Azienda Ospedaliero-Universitaria di Bologna, Bologna, Italy; 8 Department of Health Care of Moscow, Research and Practical Clinical Center of Diagnostics and Telemedicine Technologies, Moscow, Russia; 9 Department of Medical Imaging, Saint Joseph Hospital, Paris, France; 10 Department of Radiology, UZ Leuven, Leuven, Belgium; 11 Section of Radiology, Ankara Golbasi Sehit Ahmet Ozsoy State Hospital, Ankara, Turkey; 12 Department of Civil and Environmental Engineering, Stanford University, Stanford, CA USA; 13 Department of Radiology, UT Southwestern Medical Center, Dallas, TX USA

**Keywords:** Radiology, Diagnostic imaging, Artificial intelligence, Surveys and questionnaires

## Abstract

**Objectives:**

Radiologists’ perception is likely to influence the adoption of artificial intelligence (AI) into clinical practice. We investigated knowledge and attitude towards AI by radiologists and residents in Europe and beyond.

**Methods:**

Between April and July 2019, a survey on fear of replacement, knowledge, and attitude towards AI was accessible to radiologists and residents. The survey was distributed through several radiological societies, author networks, and social media. Independent predictors of fear of replacement and a positive attitude towards AI were assessed using multivariable logistic regression.

**Results:**

The survey was completed by 1,041 respondents from 54 mostly European countries. Most respondents were male (*n* = 670, 65%), median age was 38 (24–74) years, *n* = 142 (35%) residents, and *n* = 471 (45%) worked in an academic center. Basic AI-specific knowledge was associated with fear (adjusted OR 1.56, 95% CI 1.10–2.21, *p* = 0.01), while intermediate AI-specific knowledge (adjusted OR 0.40, 95% CI 0.20–0.80, *p* = 0.01) or advanced AI-specific knowledge (adjusted OR 0.43, 95% CI 0.21–0.90, *p* = 0.03) was inversely associated with fear. A positive attitude towards AI was observed in 48% (*n* = 501) and was associated with only having heard of AI, intermediate (adjusted OR 11.65, 95% CI 4.25–31.92, *p* < 0.001), or advanced AI-specific knowledge (adjusted OR 17.65, 95% CI 6.16–50.54, *p* < 0.001).

**Conclusions:**

Limited AI-specific knowledge levels among radiology residents and radiologists are associated with fear, while intermediate to advanced AI-specific knowledge levels are associated with a positive attitude towards AI. Additional training may therefore improve clinical adoption.

**Key Points:**

*• Forty-eight percent of radiologists and residents have an open and proactive attitude towards artificial intelligence (AI), while 38% fear of replacement by AI.*

*• Intermediate and advanced AI-specific knowledge levels may enhance adoption of AI in clinical practice, while rudimentary knowledge levels appear to be inhibitive.*

*• AI should be incorporated in radiology training curricula to help facilitate its clinical adoption.*

**Supplementary Information:**

The online version contains supplementary material available at 10.1007/s00330-021-07781-5.

## Introduction

Artificial intelligence (AI) and deep learning (DL) algorithms have shown a promising performance when applied to medical imaging [1–4]. AI offers substantial opportunities for radiologists, such as increasing workflow efficiency and faster and more reproducible segmentation and detection tasks [5–7]. Although AI currently dominates conferences and literature, it is still in its early phase of clinical adoption. So far, only scarce narrow task detection DL-based models have been implemented in selected centers [8].

A common recommendation for radiologists is to get involved in AI and hold matters within their own hands to avoid turf losses to industry or other specialties [9, 10]. Medicine is an industry known for trailing behind the technological advancements of other industries. Radiologists and residents with an open and proactive attitude (i.e., those who are willing to invest extra time in AI in an already full clinical schedule) can be considered *early adopters*. These proactive physicians are needed to drive the next phase so that the early majority will start using the tools and a tipping point can be reached [11]. This is crucial, because this will enable thorough validation of AI tools in clinical practice while feedback of the end-user is generated. Adoption of AI by radiologists may also prevent the dreaded scenario that data is used for financial reasons rather than improvement of patient care [12].

With impressive software results as well as unnuanced and often misleading statements in literature and the mainstream media, a general undertone of “fear of replacement,” either by computers or other disciplines, seems to have developed amongst medical students, trainees, and even radiologists, as was shown in smaller scale surveys [13–17]. Other smaller scale surveys (ranging from 69 to 270 respondents), as well as a recent larger (*n* = 675) survey, have suggested that this fear is passing and showed a positive attitude towards the topic of AI amongst medical students or radiology professionals [14, 18–22]. Only one survey (*n* = 270) investigated the existing knowledge level of radiologists pertaining to AI [20]. The current general attitude and level of knowledge of AI among residents and radiologists at large remain relatively unknown. The purpose of this large-scale international study was to investigate the existing knowledge and general attitude towards AI among international radiologists and residents, and to explore their associations.

## Materials and methods

### Questionnaire

No institutional review board approval was needed. Analysis was done with anonymized data. A web-based survey using Google Forms (Google LLC) was created consisting of 39 questions on demographics, background, social media use, awareness and existing knowledge, attitude towards AI, willingness to actively engage, AI integration in radiology training, and anticipated hurdles to AI implementation. Answers were multiple choice or open (Appendix 1). A pilot was done with 10 radiologists and residents to eliminate ambiguity [23]. The survey was then adjusted and translated by native speakers in nine languages (English, French, German, Spanish, Italian, Dutch, Czech, Russian, and Turkish).

### Participant outreach

The survey and a brief cover letter were accessible between April 18 and July 12, 2019 on www.airadiologysurvey.com. The survey was distributed through the Radiological Society of the Netherlands (NVvR, *n* = 2,002 members), the Italian Society of Medical Radiology (SIRM, *n* = 10,320 members), and the French Society of Radiology (SFR, *n* = 8,300 members) by email to all members. The NVvR and European Society of Medical Imaging Informatics (EuSoMII) featured a bulletin on their website. The Canadian Association of Radiology (CAR) promoted the survey through social media. Furthermore, the authors promoted the survey through their professional network and emails were sent to radiologists and residents within some authors’ institutions (i.e., five Dutch hospitals). In addition, 13 Canadian and 15 American program directors were approached with the request to forward the survey in their institutions. The survey was included in resident and fellow section (RFS) AI Journal Club newsletter on May 28, 2019. Additionally, the survey was featured in AuntMinnie [24] and AuntMinnieEurope [25]. The survey was also repeatedly promoted on social media (LinkedIn and Twitter) via the personal accounts of the authors and EuSoMII. The pre-defined sample size was *n* = 1,000 to allow for robust analyses and conclusions, and the survey was closed once that target was reached.

### Statistical analysis

Continuous data are presented as means with standard deviations or medians with ranges. Categorical data are presented as proportions. Univariable analysis for categorical data was done using chi-square tests and a Kruskal-Wallis test for age. Associations of independent variables with the outcomes on knowledge and attitude were assessed using multivariable logistic regression (enter method) to correct for possible confounders and/or effect modifiers. Variables (age, gender, region (European versus non-European), working in academia, scientific background, current position (resident versus radiologist), source population (SIRM, SFR, NvVR, other), professional social media use, knowledge of informatics/statistics, AI-specific knowledge, and subspecialty were selected beforehand. Age was modeled as a continuous variable; all other variables were modeled as categorical variables. For AI-specific knowledge, the categories 4 (advanced knowledge) and 5 (actively engaged in research/development) were combined for robustness, and labeled “advanced knowledge.” Category 2 was labeled “basic knowledge,” and category 3 on the 5-point scale was labeled “intermediate knowledge.” The variable “fear of replacement” was dichotomized in “yes and maybe” versus “no” for question 25 (“Do you think the diagnostic radiologist’s job is in danger due to AI?”). An open and proactive attitude towards AI was defined as readiness to use and learn about AI, willingness to collaborate with data scientists, and agreement that radiologists should take the lead. For statistical analysis, “open and proactive attitude” was dichotomized according to having answered “yes” versus “no” or “maybe” on questions 37 (Should radiologists take the lead in development of AI technology?), 33 (Would you be willing to use AI software in the clinical setting?), 34 (Would you be interested in collaborating with computer scientists or data scientists to develop an AI algorithm?), and 31 (Are you planning on learning about this topic, even if it’s not a program or CME requirement?) (see Appendix 1). Results of the logistic regression analyses are presented as adjusted odds ratios (ORs) with 95% confidence intervals (CI). Statistical analysis of the results was done in IBM SPSS Statistics for Windows (Version 24.0.: IBM Corp.). A *p* value < 0.05 was deemed statistically significant.

## Results

### Demographics

**Fig. 1:**
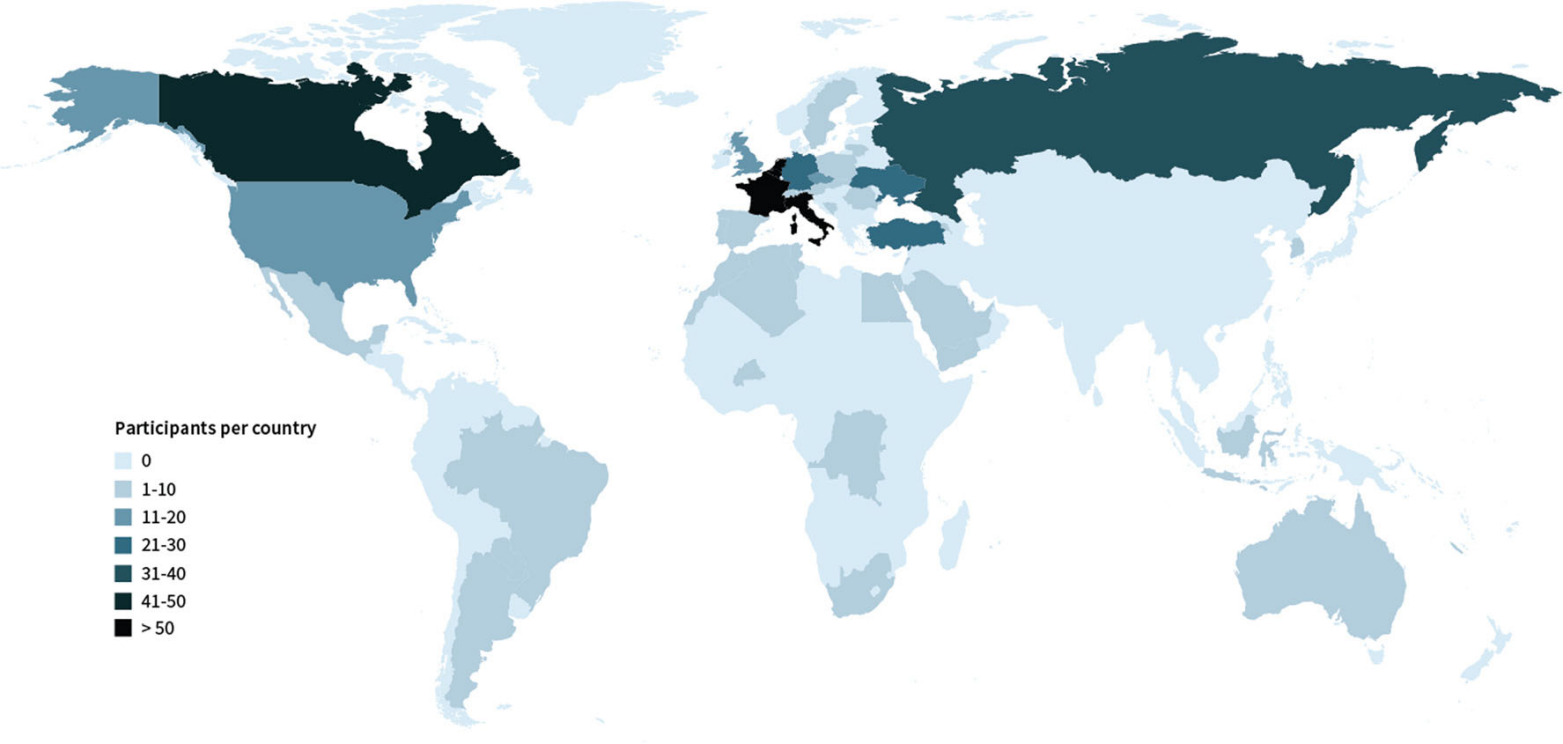
Geographic heat map of survey respondents

A total of 1,086 respondents completed the survey. Forty-five respondents were excluded because they were the not the target population (e.g., student, industry, researcher, entrepreneur) or were double entries, resulting in a final population of 1,041 respondents from 54 countries (Fig. 1). Of respondents, *n* = 272 (26%) were SIRM members, *n* = 185 (18%) SFR members, and *n* = 274 (26%) NVvR members, with response rates of 2.6%, 2.2%, and 13.7% respectively. *N* = 310 (30%) respondents were recruited through social media or personal networks.

Most respondents (*n* = 867, 83%) worked in European countries, *n* = 109 (11%) worked in non-European countries, and (*n* = 64, 6%) worked in North America/Canada. Most respondents were male (*n* = 670, 65%) and the median age was 38 (24–74) years. Respondents were working either in non-academic hospitals (*n* = 367, 35%) or in the private sector (*n* = 203, 20%), and *n* = 471 (45%) worked in an academic center. Mostly, respondents were radiologists (*n* = 692, 66%) with a median of 12 (0–44) years of experience, not including residency. *N* = 142 (35%) were residents, the majority senior (*n* = 173, 56%, defined as completed > 50% of program). Only a minority were fellows (*n* = 27, 3%).

A quarter of respondents indicated being a generalist (i.e., > 4 subspecialties chosen, *n* = 255, 25%). Of the residents and radiologists, most had one subspecialty (*n* = 513, 49%), and *n* = 273 (26%) two or more subspecialties. The most commonly listed subspecialties were abdominal imaging (*n* = 328, 32%), musculoskeletal imaging (*n* = 241, 23%), and neuroradiology (*n* = 208, 20%). Molecular/nuclear imaging was the subspecialty of *n* = 41 (4%) respondents.

The majority of respondents (*n* = 727, 70%) had no scientific background, apart from the MD degree. A minority (*n* = 171, 16%) completed a PhD. Those with a scientific background tended to work more often in academia (*p* < 0.001), independent of gender and current position. The source populations were different with respect to most characteristics, except for gender, which was evenly distributed. A summary of the respondents’ demographics stratified per source population is given in Table 1.

**Table 1:** Baseline characteristics of respondents per source population (*n* = 1,041)

Category		SIRM (*n* = 272)	SFR (*n* = 185)	NVvR (*n* = 274)	Other (*n* = 310)	Total *N* (%)	*p* value
Gender (male)		180 (69%)	112 (61%)	161 (61%)	209 (68%)	670 (65%)^a^	NS
Age (median (range)		41 (26–74)	50 (24–70)	37 (25–73)	43 (24–65)	38 (24–70)	< 0.001
Region	Africa	0 (0%)	10 (5%)	0 (0%)	4 (1%)	14 (1%)	< 0.001
	Asia	0 (0%)	3 (2%)	0 (0%)	70 (23%)	73 (7%)	
	Australia	0 (0%)	2 (1%)	3 (1%)	3 (1%)	8 (1%)	
	Europe	272 (100%)	162 (88%)	269 (98%)	164 (53%)	867 (83%)	
	North America	0 (0%)	0 (0%)	1 (0.5%)	64 (21%)	65 (6%)	
	South America	0 (0%)	8 (4%)	1 (0.5%)	5 (1%)	14 (1%)	
Type of hospital	Academic	120 (44%)	55 (30%)	99 (36%)	197 (64%)	471 (45%)	< 0.001
	Non-academic	103 (38%)	41 (22%)	168 (61%)	55 (18%)	367 (35%)	
	Private	49 (18%)	89 (48%)	7 (3%)	58 (19%)	203 (20%)	
Current position	Radiologist	196 (72%)	163 (88%)	157 (57%)	176 (57%)	692 (66%)	< 0.001
	Fellow	1 (0%)	0 (0%)	13 (5%)	13 (4%)	27 (3%)	
	Resident	75 (28%)	22 (12%)	104 (38%)	121 (39%)	322 (31%)	
Sub specialization	Abdominal	108 (40%)	64 (35%)	49 (18%)	107 (35%)	328 (32%)	< 0.001
	Musculoskeletal	79 (29%)	38 (21%)	45 (16%)	79 (26%)	214 (23%)	< 0.01
	Neuro	40 (15%)	36 (20%)	39 (14%)	93 (30%)	208 (20%)	< 0.001
	Interventional	43 (16%)	40 (22%)	41 (15%)	59 (19%)	183 (18%)	NS
	Breast	50 (18%)	4 (2%)	20 (7%)	41 (13%)	115 (11%)	< 0.001
	Cardiothoracic	56 (21%)	21 (11%)	28 (10%)	74 (24%)	179 (17%)	< 0.001
	Pediatric	23 (9%)	25 (14%)	7 (3%)	34 (11%)	89 (9%)	< 0.001
	Molecular/nuclear	2 (1%)	3 (2%)	19 (7%)	17 (6%)	41 (4%)	< 0.001
Advanced scientific background^b^	No	230 (84%)	137 (74%)	165 (60%)	195 (63%)	727 (70%)	< 0.001
	PhD	16 (6%)	14 (7%)	71 (26%)	47 (15%)	148 (14%)	
	Research fellowship	10 (4%)	20 (11%)	3 (1%)	18 (6%)	51 (5%)	
	PhD and research fellowship	3 (1%)	9 (5%)	6 (2%)	5 (2%)	23 (2%)	
	Obtaining PhD/research fellowship	13 (5%)	5 (3%)	29 (11%)	45 (15%)	92 (9%)	
Social media use (professional)	No	128 (47%)	127 (69%)	112 (41%)	110 (36%)	477 (46%)	< 0.001
	Yes	144 (53%)	58 (31%)	162 (59%)	200 (65%)	564 (54%)	
	LinkedIn	81 (30%)	33 (18%)	149 (54%)	97 (31%)	360 (64%)	< 0.001
	Twitter	14 (5%)	12 (7%)	22 (8%)	67 (22%)	115 (20%)	< 0.001
	Instagram	24 (9%)	4 (2%)	11 (4%)	60 (19%)	99 (18%)	< 0.001
	Facebook	67 (25%)	25 (14%)	22 (8%)	85 (27%)	78 (14%)	< 0.001

^a^Prefer not to say (*n* = 14)

^b^In addition to medical school

*SIRM*, Italian Society of Medical Radiology; *SFR*, French Society of Radiology; *NVvR*, Radiological Society of the Netherlands

### Knowledge of statistics/informatics

Almost half of the participants indicated to have knowledge of informatics/statistics (*n* = 504, 48%), mostly without a formal degree (*n* = 465, 45%). A university degree in informatics or statistics was observed in *n* = 29 (3%) respondents. Coding skills were indicated in *n* = 77/312 (24%, Table 2). Males were more likely to have knowledge of statistics/informatics (adjusted OR 2.39, 95% CI 1.78–3.21, *p* < 0.001) as well as having a scientific background (adjusted OR 2.29, 95% CI 1.68–3.11, *p* < 0.001). Knowledge of statistics/informatics was evenly distributed among subspecialties.

**Table 2:** Self-assessed knowledge, fear and attitude (*n* = 1,041)

Self-assessed knowledge		
Knowledge of informatics/statistics	No	537 (52%)
	Yes, no degree	465 (45%)
	Yes, degree	39 (4%) any degree 29 (3%) university level
AI-specific knowledge	0 Never heard of AI	47 (4%)
	1 Heard of AI	221 (21%)
	2 Basic knowledge	307 (30%)
	3 Intermediate knowledge	296 (28%)
	4 Advanced knowledge	111 (11%)
	5 Active research/development	57 (6%)
Coding skills (any language)^a^	None	235 (75%)
	Basic	63 (20%)
	Advanced	14 (4%)
Fear of replacement		
Do you think the diagnostic radiologist's job is in danger due to AI?	No	640 (62%)
	Yes	140 (13%)
	Maybe	261 (25%)
Career doubt		
Would you have chosen for a career as a radiologist again with your current knowledge of AI?	No	86 (8%)
	Yes	795 (77%)
	Maybe	160 (15%)
Attitude		
Should radiologist take the lead in the development of AI technology?	No	36 (4%)
	Yes	826 (79%)
	Maybe	179 (17%)
Would you be willing to use AI software in the clinical setting?	No	14 (1%)
	Yes	885 (85%)
	Maybe	142 (14%)
Would you be interested in collaborating with computer scientists or data scientists to develop an AI algorithm?	No	94 (9%)
	Yes	724 (70%)
	Maybe	223 (21%)
Are you planning on learning about this topic (i.e. AI), even if it's not a program or CME requirement?	No	63 (6%)
	Yes	780 (75%)
	Maybe	198 (19%)
Respondents with an open and proactive attitude^b^		501 (48%)

^a^This questions was only incorporated in the English, Dutch, French, Czech, German, and Russian translations (total *n* respondents = 312)

^b^Defined as having answered “yes” to all four attitude questions

### AI-specific knowledge

A minority of respondents had only heard of AI (*n* = 221, 21%, Table 2). Only having heard of AI was significantly less often observed in respondents with a scientific background (adjusted OR 0.51, 95% CI 0.34–0.78, = 0.002) as well in cardiothoracic subspecialists (adjusted OR 0.53, 95% CI 0.32–0.86, *p* = 0.01).

A minority of respondents (*n* = 168, 16%) had advanced knowledge or were actively engaged in research and/or development of AI (Table 2). Advanced knowledge or active engagement was significantly more often observed in males (adjusted OR 2.10, 95% CI 1.34–3.31, *p* = 0.001), Europe (adjusted OR 2.33, 95% CI 1.29–4.22, *p* = 0.005), academia (adjusted OR 1.80, 95% CI 1.18–2.73, *p* = 0.006), having a scientific background (adjusted OR 2.74, 95% CI 1.85–4.07, *p* < 0.001), and professional social media use (adjusted OR 2.76, 95% CI 1.82–4.19, *p* < 0.001). Participants with musculoskeletal interest (adjusted OR 0.40, 95% CI 0.23–0.69, *p* = 0.001) reported less commonly to be knowledgeable on AI.

### Fear of replacement and career doubt

Fear of replacement was found in 38% (*n* = 401, Table 2). Fear was significantly more often reported in males (adjusted OR 1.86 95% CI 1.38–2.52, *p* < 0.001) and participants with basic AI-specific knowledge (adjusted OR 1.56, 95% CI 1.10–2.21, *p* = 0.01). Fear was significantly less often reported with increasing age (adjusted OR 0.77 per 10-year interval, 95% CI 0.66–0.90, *p* = 0.002), and those with intermediate AI-specific knowledge (adjusted OR 0.40, 95% CI 0.20–0.80, *p* = 0.01) or advanced AI-specific knowledge (adjusted OR 0.43, 95% CI 0.21–0.90, *p* = 0.03). Fear was not associated with region, source population, current position, working in academia, or subspecialty.

The most common reasons for fearing replacement were predicting that the role of the diagnostic radiologist would be altered, however not being replaced (*n* = 329, 82%), suspecting full replacement (*n* = 42, 10%), and suspecting partial replacement resulting in a decline in demand for radiologists (*n* = 23, 6%).

Given the respondent’s knowledge level of AI, *n* = 86 (8%) indicated that they would have chosen a career as a radiologist again, and *n* = 160 (15%) might not have chosen a career as a radiologist again (Table 2). This career doubt was significantly associated with fear of replacement (adjusted OR 4.41, 95% CI 3.16–6.17, *p* < 0.001). Professional social media use appeared to be a protective factor against career doubt (adjusted OR 0.69, 95% CI 0.49–0.96, *p* = 0.03). Career doubt was not associated with age, gender, region, current position, working in academia, AI-specific knowledge, or subspecialty.

### Open and proactive attitude

Agreeing that radiologists should take the lead in development of AI technology (*n* = 826, 79%) was significantly more observed in males (adjusted OR 1.82, 95% CI 0.1.30–2.59, *p* = 0.001), those only having heard of AI (adjusted OR 3.62, 95% CI 1.76–7.42, *p* < 0.001), as well as having intermediate (adjusted OR 4.29, 95% CI 2.04–9.00, *p* < 0.001) or advanced AI-specific knowledge (adjusted OR 6.49, 95% CI 2.78–15.17, *p* < 0.001). Neuroradiologists disagreed significantly more often (adjusted OR 0.59, 95% CI 0.40–0.87, *p* = 0.009).

A willingness to use AI in clinical practice (*n* = 885, 85%) negatively correlated with a fear of replacement (adjusted OR 0.67, 95% 0.46–0.98, *p* = 0.04). Males were more willing to use AI in clinical practice (adjusted OR 1.77, 95% CI 1.19–2.63, *p* < 0.01). Having heard of AI (adjusted OR 2.50, 95% CI 1.24–5.06, *p* = 0.01), and having intermediate (adjusted OR 4.80, 95% CI 2.22–10.38, *p*< 0.001) or advanced AI-specific knowledge (adjusted OR 5.38, 95% CI 2.15–13.51, *p* < 0.001) were associated with a willingness to use AI in clinical practice as well.

Basic knowledge of AI was a negative predictor for wanting to collaborate with data scientists (adjusted OR 0.66, 95% CI 0.46–0.96, *p* = 0.03). Male gender (adjusted OR 1.49, 95% CI 1.08–2.05, *p* = 0.01), professional social media use (adjusted OR 1.58, 95% CI 1.17–2.14, *p* = 0.003), and intermediate (adjusted OR 2.22, 95% CI 1.09–4.51, *p* = 0.03) or advanced knowledge (adjusted OR 7.12, 95% CI 2.90–17.47, *p* < 0.001) were positive predictors.

Positive predictors for interest to learn about AI (*n* = 780, 75%) were having heard of AI (adjusted OR 2.27, 95% CI 1.15–4.52, *p* = 0.02), as well as having intermediate (adjusted OR 6.22, 95% CI 3.00–12.88, *p* < 0.001) or advanced AI-specific knowledge (adjusted OR 15.29, 95% CI 6.07–38.50, *p* < 0.001), independent of age, gender, and other demographics.

Almost half of the respondents appeared to have an open and proactive attitude towards AI (*n* = 501, 48%, Table 3). Positive predictors for an open and proactive attitude were male gender (adjusted OR 1.77, 95% CI 1.29–2.42, *p* < 0.001), only having heard of AI (adjusted OR 4.78, 95% CI 1.78–13.32, *p* = 0.002), intermediate (adjusted OR 11.65, 95% CI 4.25–31.92, *p* < 0.001) or advanced AI-specific knowledge (adjusted OR 17.65, 95% CI 6.16–50.54, *p* < 0.001). Negative predictors for an open and proactive attitude were increasing age (adjusted OR 0.78 per 10-year interval, 95% CI 0.66–0.93, *p* = 0.006) and basic AI-specific knowledge (adjusted OR 0.58, 95% CI 0.41–0.83, *p* = 0.002). Having an open and proactive attitude was not associated with region, source population, working in academia, current position, subspecialty, or fear of replacement. All predictors for an open and proactive attitude are listed in Table 3.

**Table 3:** Predictors for an open and proactive attitude in a multivariable logistic regression model (*n* = 1,041)

Predictor		Odds ratio^a^	CI^b^	*p* value
Male		1.77	1.29–2.42	< 0.001+
Age (per 10-year interval)		0.78	0.66–0.93	0.006
Professional social media use		1.64	1.23–2.18	0.001
Scientific background		1.63	1.18–2.45	0.003
Knowledge of informatics/statistics		1.48	1.11–1.97	0.008
Heard of AI		4.78	1.78–13.32	0.002
AI-specific knowledge	Basic	0.58	0.41–0.81	0.002
	Intermediate	11.65	4.25–31.92	< 0.001
	Advanced knowledge or active engagement	17.65	6.16–50.54	< 0.001

^a^Adjusted for region, source population, working in academia, resident, subspecialty, and fear of replacement

^b^*CI*, confidence interval

## Discussion

This large (*n* = 1,041) survey of radiologists and residents showed that intermediate and advanced AI-specific knowledge levels were associated with an open and proactive attitude towards AI. Radiologists and residents with basic knowledge levels, on the other hand, had a significantly less open and proactive attitude towards AI. This may indicate that increased AI-specific knowledge enhances adoption of AI in clinical practice, while basic knowledge levels may be inhibitive. Fear of replacement by AI still exists in the radiology community, as this was reported by 39% (*n* = 401). Career doubt was reported in 23% (*n* = 246). An open and proactive attitude towards AI was observed in almost half of respondents (48%, *n* = 501). We found a significant independent association between an open and proactive attitude towards AI and male gender, younger age, scientific background, professional social media use, and knowledge of informatics/statistics. This indicates that radiology residents and radiologists inherently have a positive attitude towards the recent technological development of artificial intelligence, especially those who still have most of their career ahead of them, and those who are naturally more inclined to be interested in science and/or technology. Interestingly, basic AI-specific knowledge was independently associated with both fear of replacement and being less likely to have an open and proactive attitude towards AI. A possible explanation for this could be that those who have had only limited exposure to AI may not be entirely informed about the limitations of AI, and hence fear job replacement. As they have a less nuanced frame of reference regarding AI, and may perceive the technology as advanced than their own skillset, it is possible that this group does not realize that radiologists can be key players in development, validation, and implementation into clinical practice, and therefore appear less open and proactive. Unfortunately, underlying reasons for this observation cannot be deducted from this study.

The largest (*n* = 675) published survey to date regarding AI in radiology, held by the European Society of Radiology (ESR) among its members in November 2018, had a somewhat different focus. The researchers investigated the expected impact of AI on different aspects of the radiologists’ daily job (e.g., subspecialties and modalities) rather than attitude and knowledge, although impact on job opportunities was assessed as well [19]. Also, the ESR survey targeted a slightly different population, including fewer residents, i.e., 4% vs. 31% in our study, and slightly more academic participants, 51% vs. 45% respectively. Therefore, the surveys can be seen as complimentary. Furthermore, in our study, the large data set allows for robust analyses regarding predictors for a radiology resident and radiologists’ AI-specific knowledge and attitude, something that has not been done before. In the ESR survey, a generally favorable attitude towards AI was observed, in line with our survey. In their survey, 42% foresaw a decrease of job opportunities, comparable to the 39% of participants fearing for (partial) replacement in our study. In the survey by Waymel et al conducted in early 2019 with 270 radiology residents and radiologists responding, the knowledge and willingness to learn was also assessed [20]. They reported that 73% had insufficient knowledge of AI, while 23% had basic knowledge as defined by the authors. Our study showed higher levels of AI-specific knowledge, with at least basic AI-specific knowledge in 76% (*n* = 771/1,041) of respondents.

In our study, the response rate was low, namely 3.9% for the combined society populations, slightly higher than reported in the ESR survey (2.8%) [19]. This introduces selection bias, a common problem encountered in questionnaire research, especially when a convenience sample is taken [26]. Therefore, the true level of knowledge and the proportion having an open and proactive attitude is most likely lower, and the level of fear may be higher. Nevertheless, the associations found between knowledge and attitude were significant and consistent throughout the analysis and therefore should hold true for the domain of interest. The determination for the level of knowledge in this survey was entirely subjective, a problem inherent to this method of research. The levels of knowledge endorsed by the respondents are a mere indication of the self-perceived knowledge, not an absolute measure. Furthermore, the definition of an “open and proactive attitude” is also a subjective measure defined by the authors. Answering yes to the four questions of interest does not guarantee that a respondent will invest extra time to help adoption of AI in clinical practice; however, it does reflect an overall positive attitude. North America is underrepresented in this survey, potentially due the tendency of their societies (e.g., RSNA) not to participate in third-party initiatives. Also, South America, Asia, and Africa were not systematically included in this survey through official channels. Therefore, the results should be interpreted as a reflection of the opinion of the radiology community in western society, mainly Europe.

This large international survey shows that limited AI-specific knowledge levels among radiology residents and radiologists are associated with fear, while intermediate to advanced AI-specific knowledge levels are associated with a positive attitude towards AI and therefore may improve clinical adoption. These findings underline that AI should be incorporated into radiology training curricula and post-academic training.

## Supplementary Information

ESM 1 (DOCX 22 kb)

## Declarations

### Guarantor

The scientific guarantor of this publication is Merel Huisman.

### Statistics and biometry

One of the authors has significant statistical expertise.

### Methodology

• cross sectional study

• multicenter study
