# Supplementary material for: An international survey on AI in radiology in 1,041 radiologists and radiology residents part 1: fear of replacement, knowledge, and attitude
Source: Eur Radiol. 2021 Mar 20;31(9):7058–66. doi: 10.1007/s00330-021-07781-5 (PMC8379099; doi:10.1007/s00330-021-07781-5)
Supplement: Supplementary file 1 — (DOCX 22 kb) [file 330_2021_7781_MOESM1_ESM.docx]

# Appendix

Appendix 1. **International Survey on AI in Radiology in English**

There were 14 respondents for the Czech version, 274 for the Dutch version, 167 for the English version, 189 for the French version, 26 for the German version, 285 for the Italian version, 55 for the Russian version, 7 for the Spanish version and 24 for the Turkish version.

**Demographics**

1. In which country do you work?
2. What is your gender?
   1. Male
   2. Female
   3. Prefer not to say
3. What is your age?

**Background**

1. In which hospital are you currently working?
2. What type of hospital is this?
   1. Academic Hospital
   2. Non-Academic(teaching) Hospital
   3. Private Hospital
   4. Other (free text)
3. Did you complete a PhD-program or research fellowship?
   1. Yes, I completed a PhD-program
   2. Yes, I completed a research fellowship
   3. Yes, I completed both a PhD-program and a research fellowship
   4. No, but I'm currently obtaining one
   5. No
4. What is your current position?
   1. Resident in Radiology
   2. Clinical Fellow in Radiology
   3. Attending Radiologist
   4. Other

**Resident in Radiology**

1. How many years is the official duration of the residency program in your country?
2. What year of your residency program are you in?
3. What is your intended / chosen subspecialty?
   1. Abdominal Imaging
   2. Breast Imaging
   3. Cardiothoracic Imaging
   4. Interventional Radiology
   5. Musculoskeletal Imaging
   6. Neuroradiology
   7. Nuclear Medicine
   8. Pediatric Radiology
   9. Other

**Attending Radiologist / Clinical Fellow**

1. Since when are you an attending radiologist?
2. Did you complete a clinical fellowship or are you currently a clinical fellow?
   1. Yes
   2. No
3. Which clinical fellowship(s) are you doing or did you complete?
   1. Abdominal Imaging
   2. Breast Imaging
   3. Cardiothoracic Imaging
   4. Interventional Radiology
   5. Musculoskeletal Imaging
   6. Neuroradiology
   7. Nuclear Medicine
   8. Pediatric Radiology
   9. Other

**Social Media**

1. Do you use social media for professional purposes?
   1. Yes
   2. No
2. What social media do you use for professional purposes (multiple answers possible)?
   1. Twitter
   2. LinkedIn
   3. Facebook
   4. Instagram
   5. Other
   6. None

**Awareness and existing knowledge**

1. Have you heard of artificial intelligence/deep learning/machine learning?
   1. Yes
   2. No
2. If yes, how deep is your knowledge of artificial intelligence/deep learning/machine learning (Likert-scale)?

1 (Heard of it) 2 3 4 5 (Actively engaged in research/development)

1. Do you have a background in informatics / statistics?
   1. Yes, I have a degree in informatics / statistics
   2. Yes, but I have no degree in informatics / statistics
   3. No

**Degree in informatics / statistics**

1. What degree in informatics / statistics do you have?

**Awareness and existing knowledge (2)^a^**

1. Are you capable of programming?
   1. Yes, basic coding skills
   2. Yes, advanced coding skills
   3. No coding skills
2. Is your hospital currently using AI-software?
3. Yes
4. No
5. I don’t know
6. What kind of AI-software is your hospital using?

**Attitude towards the topic**

1. Do you think AI will alter the future of radiologists?
2. Yes
3. No
4. Maybe
5. If yes/maybe, on what term do you think radiologists will notice the effects of AI?
6. < 5 years
7. 5 - 10 years
8. 10 - 20 years
9. > 20 years
10. Do you think the diagnostic radiologist's job is in danger due to AI?
11. Yes
12. No
13. Maybe

**Attitude towards the topic** (1)

1. Why do you think the diagnostic radiologist's job is not in danger?
2. AI is just a hype
3. The role of diagnostic radiologists will alter, but AI will not replace them
4. Other

**Attitude towards the topic** (2)

1. Why do you think the diagnostic radiologist's job may be in danger?
2. AI will fully replace diagnostic radiologists
3. The role of diagnostic radiologists will alter, but AI will not replace them
4. Other

**Attitude towards the topic** (3)

1. Do you believe AI can help to improve diagnostic radiology?
2. Yes
3. No
4. Maybe
5. If yes/maybe, how do you think AI can help diagnostic radiology on the long term? (Multiple answers possible)
6. AI will assist radiologists with image analysis (e.g. as a second reader)
7. AI will partially replace radiologists by performing part of the image analysis (e.g. human reader only evaluates abnormal chest X-rays)
8. AI will assist radiologists by optimizing workflow
9. AI will fully replace radiologists
10. Other
11. Would you have chosen for a career as a radiologist again with your current knowledge of AI?
12. Yes
13. No
14. Maybe

**Willingness to actively engage**

1. Are you planning on learning about this topic, even if it's not a program or CME requirement?
2. Yes
3. No
4. Maybe
5. If yes/maybe, how are you planning on learning or did you learn about this topic? (Multiple answers possible)
6. Scientific literature (E.g. pubmed, arXiv)
7. Conferences/specialty courses
8. E-learning platform (E.g. Coursera/EdX)
9. Social media (Twitter, LinkedIn, Facebook, Youtube, etc)
10. Online articles (E.g. Medium.com, ai.myesr.org)
11. Other
12. Would you be willing to use AI software in the clinical setting?
13. Yes
14. No
15. Maybe
16. Would you be interested in collaborating with computer scientists or data scientists to develop an AI algorithm?
17. Yes
18. No
19. Maybe

**AI integration in radiology training**

1. Should AI education become part of residency programs?
2. Yes
3. No
4. Maybe
5. Should imaging informatics / AI become a subspecialty?
6. Yes
7. No
8. Maybe
9. Other
10. Should radiologists take the lead in development of AI technology?
11. Yes
12. No
13. Maybe

**Hurdles to AI implementation**

1. What would be the biggest hurdle that has to be taken before AI can be implemented in clinical practice?
2. High costs of AI software development
3. High costs of AI software
4. Lack of trust in AI by clinicians / staff / management
5. Lack of knowledge / expertise of clinicians / staff / management
6. Lack of high quality image data
7. Lack of high quality image labels
8. Lack of generalizability of AI software
9. Ethical / legal issues, e.g. who is accountable for the results
10. Limitation in digital infrastructure in hospital
11. Other
    1. These questions were only incorporated in the English, Dutch, French, Czech, German and Russian translations (total n respondents= 312)
